# Underutilization of Sodium-Glucose Cotransporter 2 (SGLT-2) Inhibitors in Diabetic Nephropathy Patients in Government-Run Hospitals in India

**DOI:** 10.7759/cureus.38676

**Published:** 2023-05-07

**Authors:** Jay Maradia, Shirish S Joshi, Amitoj Sohal

**Affiliations:** 1 Pharmacology and Therapeutics, Seth Gordhandas Sunderdas Medical College and King Edward Memorial (KEM) Hospital, Mumbai, IND

**Keywords:** empagliflozin, canagliflozin, dapagliflozin, public hospital, real-world analysis

## Abstract

The editorial discusses the beneficial effects of sodium-glucose cotransporter-2 (SGLT-2) inhibitors in patients with diabetic nephropathy and their underutilization in government hospitals in India for the treatment of diabetic nephropathy. The authors provide a comprehensive analysis of various factors contributing to the under-prescription of these medications, including lack of awareness and education among healthcare professionals, limited availability and accessibility of medications, high cost, and poor adherence to evidence-based guidelines. Addressing these factors through education, research, and affordable pricing and reimbursement policies may help improve the appropriate prescription of SGLT-2 inhibitors in government hospitals in India.

## Editorial

Seetharaman et al. recently published a comprehensive, real-world, single-center, drug utilization study that evaluated the prescribing patterns, World Health Organization (WHO) core prescribing indicators and adverse drug reactions (ADRs) encountered in non-dialysis patients with diabetic nephropathy in a public hospital in India. It also discussed the practice of prescribing anti-diabetics, anti-hypertensives, anti-microbials and other related drugs to the patients based on local and international guidelines [1].

Sodium-glucose cotransporter-2 (SGLT-2) inhibitors are a class of oral glucose-lowering agents that have demonstrated benefits in the management of diabetic nephropathy in various multi-center trials. These agents have been shown to reduce the risk of progression of chronic kidney disease, decrease albuminuria, and improve cardiovascular outcomes. The Perkovic et al. trial showed a significant risk reduction (30% reduction in relative risk compared to placebo) in end-stage kidney disease and cardiovascular events in patients with type 2 diabetes and chronic kidney disease treated with canagliflozin, an SGLT-2 inhibitor [2]. The Heerspink et al. trial demonstrated that dapagliflozin reduced the risk of kidney failure (hazard ratio, 0.61; 95% confidence interval [CI], 0.51 to 0.72; P<0.001), cardiovascular death, and all-cause mortality in patients with chronic kidney disease, regardless of the presence of diabetes [3]. The Herington et al. trial showed a reduction in the risk of end stage kidney disease (hazard ratio, 0.72; 95% confidence interval [CI], 0.64 to 0.82; P<0.001) and cardiovascular events with empagliflozin in patients with chronic kidney disease and type 2 diabetes [4]. Given the consistent benefits seen across these trials, SGLT-2 inhibitors are an important treatment option in clinical practice for patients with diabetic nephropathy.

Still there is underutilization of SGLT-2 inhibitors particularly in India. In the Seetharaman et al. study only eight out of 253 patients were prescribed SGLT-2 inhibitors [1]. There are several reasons for the underutilization of SGLT-2 inhibitors in India, particularly in government hospitals. We tried to provide a comprehensive analysis stating various factors that may contribute to the issue.

One of the main reasons for under-prescribing is the lack of awareness and education among healthcare professionals regarding the benefits and appropriate use of SGLT-2 inhibitors. This is compounded by the fact that there is a significant variation in the availability and accessibility of these medications across different regions of India, with some government hospitals not stocking them at all [1]. The other important reason for this could be the poor personal hygiene of most of the patients attending the hospital, due to which they were predisposed to urinary tract infections [1]. Another factor contributing to under-prescribing is the cost of SGLT-2 inhibitors, which can be prohibitive for patients in government hospitals who often have limited financial resources [5]. Finally, there is a lack of adherence to evidence-based guidelines for the management of diabetic nephropathy patients in government hospitals in India. This is due to the limited resources available for continuing medical education and training for healthcare professionals [5].

The relative importance of these factors and their interplay can vary depending on the specific context of government hospitals in India. Addressing these factors through education, research, affordable pricing and reimbursement policies may help increase the appropriate prescription of SGLT-2 inhibitors in government hospitals in India.

In conclusion, under-prescribing of SGLT-2 inhibitors in government hospitals in India is a complex issue with multiple contributing factors. Improving awareness and education among healthcare professionals, increasing the availability and accessibility of these medications, and adherence to evidence-based guidelines are all necessary steps to address this problem and improve the management of diabetic nephropathy patients in India.
